# Immunosuppression in kidney transplant recipients with COVID-19 infection – where do we stand and where are we heading?

**DOI:** 10.1080/0886022X.2021.1876730

**Published:** 2021-01-24

**Authors:** Ahmed Daoud, Ahmad Alqassieh, Duaa Alkhader, Maria Aurora Posadas Salas, Vinaya Rao, Tibor Fülöp, Karim M. Soliman

**Affiliations:** a Nephrology Unit, Internal Medicine Department, Kasr Alainy School of Medicine, Cairo University, Cairo, Egypt; b Department of Surgery, Medical University of South Carolina, Charleston, SC, USA; c Department of Medicine, Division of Nephrology, Medical University of South Carolina, Charleston, SC, USA; d Medicine Service, Ralph H. Johnson VA Medical Center, Charleston, SC, USA

**Keywords:** COVID-19; kidney transplant; bamlanivimab; immunosuppression; ivermectin; CNI.

## Abstract

The appropriate immunosuppressive regimen in kidney transplant recipients with severe acute respiratory syndrome coronavirus-2 (SARS-CoV-2/COVID-19) infection remains unclear. The impact of direct virus injury complicated by dysregulated hyperimmune response with overwhelming release of various cytokines in COVID-19 infected subjects contributes to the complexity of management. The largest concern of the practicing clinicians at current time is how to tailor maintenance immune-modulating therapy during active viral infection and the efficacy of the soon-to-be upcoming immunization for COVID-19. This targeted review aims to cover most of the current evidence on the effect of key maintenance immunosuppressive agents in COVID-19 infection and proposes a line of management to specific scenarios on this very rapidly evolving subject.

## Introduction

The choice of immunosuppressive drugs in solid organ transplant recipients with severe acute respiratory syndrome coronavirus 2 (SARS-CoV-2), the virus causing COVID-19 infection, remains unclear [1]. The combined impact of direct virus injury and dysregulated hyperimmune response with the aberrant release of various cytokines in COVID-19 infected subjects adds to the complexity of management [2]. Various research adopted the use of various immunosuppressive agents to modulate the host’s immune response in COVID-19 infected patients. As an example, some evidence suggests that lymphopenia is a risk factor for COVID-19 infection – the former being the mode of action of mammalian target of rapamycin [mTOR] inhibitors. However, more recent experimental studies suggest that mTOR inhibitors carry an anti-viral activity against SARS-CoV-2 [3]. This targeted review aims to cover most of the current evidence of the effect of different maintenance immunosuppressive agents in kidney transplant recipients (KTRs) during COVID-19 infection and proposes a line of management.

### Glucocorticoids

There is still some debate regarding the ideal management of glucocorticoid therapy in COVID-19 infected organ transplant recipients. Most clinicians opt to continue glucocorticoids [4]. This practice is supported by the RECOVERY trial demonstrating reduced mortality with dexamethasone therapy in hospitalized non-transplant COVID-19 patients receiving respiratory support [5]. On the contrary, some studies showed evidence of possible harm of corticosteroid treatment in SARS patients [6–10]. The potential risk of glucocorticoid therapy in COVID-19 patients include immune suppression, impaired viral clearance and higher plasma viral load and epithelial shedding. On the other hand, the key side effects of glucocorticoids including diabetes, psychosis and avascular necrosis of femoral head is a time-dependent function and unlikely to make an impact unless long-term maintenance dose changed significantly or compounded by co-morbid events [7–11]. Somewhat surprisingly, some transplant nephrologists prefer glucocorticoid-sparing maintenance immunosuppression in case of COVID-19 infection [6]. Published cases of SARS-CoV-2 in KTR showed variable mortality and morbidity outcomes of high dose steroid therapy combined with reduced or discontinued calcineurin inhibitors and antiproliferative immunosuppression [12–15]. The benefit of glucocorticoid therapy is possibly dependent on appropriate dosing and timing, and current evidence shows that its benefits outweigh risks [5].

### Calcineurin inhibitors (CNIs)

Several guidelines recommend withdrawal of CNI maintenance immunosuppression in case of SARS-COV-2 infection [16,17]. Given its strong immunosuppressive effect, CNIs may result in an aggressive life-threatening COVID-19 infection. On the contrary, CNIs have been proposed to have an antiviral effect and both cyclosporine A and tacrolimus were found to inhibit the *in vitro* replication of various coronaviruses including SARS-CoV at low non-cytotoxic concentrations [18,19]. A genome-wide analysis identified immunophilins as interaction partners of CoV proteins. Inhibition of cyclophilins by cyclosporine A was found to block the replication of all types of CoVs, including SARS-CoV [20]. Moreover, tacrolimus treatment and knock down of FK-506 binding proteins blocked SARS-CoV replication *in vitro* [18]. CNIs may also have a role in ameliorating COVID-19 induced cytokine storm. CNIs inhibit T-cell activation and suppress cytokines (interleukin (IL)-2, IL-4, tumor necrosis factor-α and interferon-γ) that mediate the cytokine storm [21]. The available data are not strong enough to advocate the use of CNIs as a treatment for COVID-19 infection. However, the data may suggest continuing at least a low dose CNI maintenance immunosuppression in KTR [22]. We are awaiting the results of the Spanish clinical trial to evaluate methylprednisolone pulses and tacrolimus in patients with COVID-19 lung injury (TACROVID) trial to find out whether tacrolimus may have a therapeutic role in COVID-19 nontransplant patients [23].

Some clinicians suggest that switching from tacrolimus to cyclosporine A may be a suitable option in COVID-19 infected KTRs [24]. According to a published case report, a 45-year-old male KTR who was solely on mycophenolate mofetil (MMF) maintenance immunosuppression, was cured from COVID-19 infection after switching from MMF to cyclosporine A, the latter justified by the authors to have an *invitro* anti-viral effect [25]. A small cohort showed better outcomes in KTRs switched from tacrolimus to cyclosporine A compared to KTRs treated with CNI dose reduction [26]. However, due to limited number of patients enrolled, these findings need to be interpreted cautiously. Clinicians should be aware of drug interactions among medications used to treat COVID-19 and transplant immunosuppression. Protease inhibitors (such as lopinavir/ritonavir) are enzyme inhibitors that inhibit cytochrome P450 enzyme 3 A (CYP3A). The coadministration of these drugs with CNIs may result in higher CNI blood levels and increased CNI toxicity. CNI dose reduction and prolonged dosing intervals may be needed in KTRs treated with lopinavir/ritonavir [27,28]. Chloroquine therapy, although with no proven benefit in COVID-19 patients yet still used in this cohort, may result in up to three-fold increase in cyclosporine A levels [29,30]. This interaction is not seen with tacrolimus [28].

### Anti-proliferative medication: MMF and mycophenolic acid (MPA)

A common practice is to withdraw MMF in KTRs with active infections including COVID-19 [31]. However, there is evidence that MMF and MPA inhibit replication of corona viruses *in vitro* [32–35], including SARS-CoV-2 [36]. On the other hand, *in vivo* studies suggest that MPA may cause more harm than benefit in case of Middle East respiratory syndrome- corona virus (MERS-CoV) infection [11, 37]. To date, it is advised to reduce the dose or discontinue the use of anti-proliferative medications in severe COVID-19 infection [1].

### Belatacept

Belatacept blocks the costimulatory B7-CD28 signal needed for T-cell activation and represents an alternative agent to CNI-based maintenance immunosuppressive therapy. It is thought that it may mitigate the cytokine storm resulting in mild clinical course of COVID-19 infection [38]. A valid concern is that it may impair the T-cell mediated antiviral immune responses [39]. The clinical course of COVID-19 infected KTRs on belatacept varied from mild to severe requiring ICU admission and the effect of its use in KTRs remain poorly defined [38,39].

### mTOR inhibitors

mTOR inhibitors have been proposed to encompass a potential anti-COVID-19 effect by hindering the effect of IL-37 and IL-38 in the inflammatory state [3]. Furthermore, previous studies have revealed the association between obesity and the mTOR pathway [40,41]. Obesity, a known risk factor for COVID-19, stimulates chronic hyperactivation of mTOR activity in multiple tissues [40, 42]. In humans, high ribosomal protein S6 kinase (S6K) activity and overphosphorylation of translation suppressor 4-Eukaryotic binding protein (4EBP), both have been described in obesity [42,43]. Additionally, accelerated adipogenesis and obesity have been reported in mice presenting a disrupted 4EBP gene [44]. Hence; it has been suggested that targeting the mTOR pathway carries a potential for obesity treatment, thus mitigating the risk of COVID-19. Consistently, S6K knockout mice are found resistant to obesity owing to an impaired biosynthesis pathway downstream of mTORC1 [40].

### Antiviral medication

Remdesivir is the first anti-viral agent approved by the Food and Drug Administration (FDA) for the treatment of COVID-19. The Adaptive COVID-19 Treatment Trial (ACTT-1) study showed equivocal results regarding remdesivir vs. placebo in COVID-19 infected subjects, hence; it is recommended to be used in combination with dexamethasone. The reason for coadministering remdesivir and dexamethasone in critically ill patients, is that the former may prevent the steroid-related delay in viral clearance proven in some studies [7,45]. Therefore, for critically ill patients requiring supplemental oxygen, the US National Institute of Health (NIH) guidelines recommend in addition to dexamethasone, treatment with remdesivir at a dose of 200 mg IV for one day followed by 100 mg IV once daily for four days or until hospital discharge [46]. However, side effects of remdesivir including increased level of serum transaminase and accumulation of drug vehicle cyclodextrin in patients with kidney dysfunction need to be considered and closely monitored [46]. Favipiravir is a Japan-based antiviral agent metabolized in the liver by aldehyde oxidase, hence; drug-drug interaction with CNIs is less of a concern. Data supporting favipiravir efficacy in KTRs infected with COVID-19 remain limited. A phase 2 preventive trial evaluating the safety and efficacy of favipiravir in COVID-19 patients was granted by the FDA in August 2020.

### Ivermectin

Despite not FDA approved, to date, there are more than 15 peer-reviewed published articles showing high efficacy of the anti-parasitic agent ivermectin in prophylaxis and treatment of all stages of COVID-19 infection [47]. *In vitro*, ivermectin administered to Vero-hSLAM cells 2 h after SARS-CoV-2 infection showed ∼5000-fold reduction of viral RNA after 48 h [48]. Its anti-viral effect is thought to be mediated through the inhibition of importin α/β-mediated nuclear transport of SARS-CoV-2 proteins [48]. Proposed dose is 0.2 mg/kg for 4–5 days. Caution is advised when using ivermectin with CNIs, the former being a known cytochrome P450 inducer, potentially altering CNI drug levels.

### Tocilizumab

Few studies have shown the potential effect of tocilizumab, an IL-6 receptor antagonist, in controlling cytokine storm in patients with COVID-19 [49]. Blocking formation of cytokines may reduce overall inflammatory response and severity of infection, another potential way to tackle the effect of the virus [49]. The dose is 4–8 mg/kg administered as a single 60-min intravenous infusion every four weeks [50]. Current data support the use of tocilizumab in severe critically ill cases requiring oxygen supplementation.

### Bamlanivimab

Bamlanivimab is a neutralizing monoclonal antibody that targets spike protein of SARS-CoV-2 and aimed to block the attachment and entry of the virus into human cells [51]. On 9 November 2020, the FDA issued an emergency use authorization for bamlanivimab for the treatment of mild-to-moderate COVID-19 in adult and pediatric patients. Bamlanivimab is not yet approved for severe hospitalized cases considering possible worsening of clinical condition [52]. The FDA authorization was based on the results of the ‘blocking viral attachment and cell entry with SARS-CoV-2 neutralizing antibodies (BLAZE-1)’ trial, a phase-2 randomized, double-blind, placebo-controlled clinical trial published in October 2020 in New England Journal of Medicine [51]. The study included 465 non-hospitalized adults with mild to moderate COVID-19 symptoms [51]. Bamlanivimab was administered to patients within three days of the first^t^ positive SARS-CoV-2 viral test. According to the administered dose of bamlanivimab, patients were divided into four groups (G-1: 700 mg dose, G-2: 2800 mg dose, G-3: 7000 mg dose and G-4: placebo group). By day 3 and 11 of the first administered dose, all groups including placebo, showed a continuous trend toward viral clearance. G-2 had the biggest difference from placebo and lowest viral count as compared to G-1 and G-3. Authors concluded that the drug is safe and potentially effective in mild-moderate COVID-19 patients [51]. With no published data on transplant patients yet, it has been used off-label in some transplants centers in the US with promising results.

### Plasma exchange and convalescent plasma

The main argument that supports performing plasma exchange for COVID‐19 patients is the elimination of COVID-19 viral particles. COVID-19 viral particles have a diameter of 60–140 nm, large enough to be eliminated with double filtration plasmapheresis [53]. Additionally, removal of cytokines that mediate the cytokine storm in COVID‐19 patients is a valid rationale [53,54]. While undergoing plasma exchange, replacing with convalescent plasma is a theoretical promising therapeutic modality [53]. Some studies have adopted this theory in management of COVID-19 infected patients on immunosuppression [55]. Plasma exchange is used for seven to 14 sessions with potential favorable outcome [56].

### Extracorporeal therapy

Ronco et al. [57], reviewed the potential beneficial role of extracorporeal therapy in sepsis, cytokine storm and COVID-19 infected patients. In COVID-19 patients, it has been postulated that extra fluid, cytokines and endotoxins removal by extracorporeal therapy all have a potential survival benefit [58]. Proposed approaches for cytokine removal include direct haemoperfusion using a neutro-macroporous sorbent; plasma adsorption on a resin after plasma separation from whole blood; hollow fiber filters with adsorptive properties; and high-dose continuous kidney replacement therapy (CKRT) with medium-or high cutoff membranes [57]. Controversial results have developed from diverse set of definitions as regards to timing of CKRT initiation and, hence; leading to biased conclusions. The effect of early vs. delayed initiation of RRT on Mortality in critically ill patients with AKI (ELAIN) randomized controlled trial defined ‘early‘ as 8 h following diagnosis of acute kidney injury (AKI) stage 2 (group 1) and ‘late’ as 12 h following diagnosis of AKI stage 2 (group 2) [59]. The authors found significantly lower mortality rate within 90 days in group 1 as compared to group 2 [59]. In contrast, Barber et al. [34] failed to show a benefit of ‘early’ start of CKRT. The latter study defined ‘early’ as 12 h after development of AKI stage 3. Not only were the aforementioned studies diverse in definitions of timing but also for AKI staging, making the comparison invalid. In COVID-19 patients, it has been postulated that levels of IL-6 > 24.3 pg/mL and D-dimer >0.28 μg/L were predictive of development of severe viral pneumonia (93.3% sensitivity and 96.4% specificity) [58]. The half-life of the inflammatory mediators and cytokines is only a few minutes [58]. Moreover, once the inflammatory cascade has begun, end-organ damage already has taken place with newly generated cytokines less relevant to clinical outcomes [60]. If we only have few minutes to hit the target, then few hours delay in starting CKRT is certainly a target-miss. In addition, the late and late start of CKRT is both representing established failures. We are in need to uniform our definitions of ‘early’ vs. ‘late’ initiation of CKRT to standardize the way of practice hoping to land on an informed decision.

### Vaccination and unexplored questions

With the upcoming availability of COVID-19 vaccines, urgent understanding is needed whether to adjust immunomodulation therapy post-immunization [61]. There are six major types of COVID-19 vaccines currently under development worldwide. These vaccines are developed from different viral components including protein subunit, recombinant, live attenuated, inactivated, nucleic acid-based and virus-like particles, and hence all acting differently [61]. On the horizon and expected to be available by the publication of this article, the two promising vaccines developed by US Moderna and BioNTech-Pfizer claim more than 94% efficacy and were developed by a unique novel technology [62]. Both vaccines depend on messenger ribonucleic acid (mRNA), at different sequence, carrying signals to the recipient’s immune system to develop immunity to coronavirus infection [62]. Additionally, the vaccine developed by AstraZeneca using the genetically altered adenovirus with addition of coronavirus protein to enhance host’s immune response showed a 90% efficacy. Reported side effects in immunocompetent hosts include pain at injection site (60%), fever (50%), headache (42%), fatigue (28%) and arthralgia (24%) [62]. However, no SARS-CoV-2 vaccine trial has enrolled immunocompromised subjects [62]. The current standard of practice for immunization is to administer inactivated or killed vaccines (such as flu vaccines) without any interruption in the immunosuppressive regimen. Xia et al.[63], in a phase 2 trial, reported favorable outcome and mild side effects of an inactivated Chinese vaccine used in immunocompetent hosts against SARS-CoV-2. In case of mRNA/DNA vaccines, theoretical concerns for their use in immunocompromised subjects include a hyper-inflammatory response and autoimmune syndromes related to increased type-I interferon [64]. These effects have been previously observed in animal models with mRNA vaccines, but not in humans [65]. Another concern is the attenuated or absent response to the mRNA/DNA vaccines in this immunocompromised cohort for about 1–6 months after initial induction therapy. Safety and immune response to these novel vaccines in immunocompromised hosts remain unclear and unpredicted. What we understand is that patients might benefit from vaccination before undergoing solid organ transplantation [62].

### Proposed management

**Figure 1.**
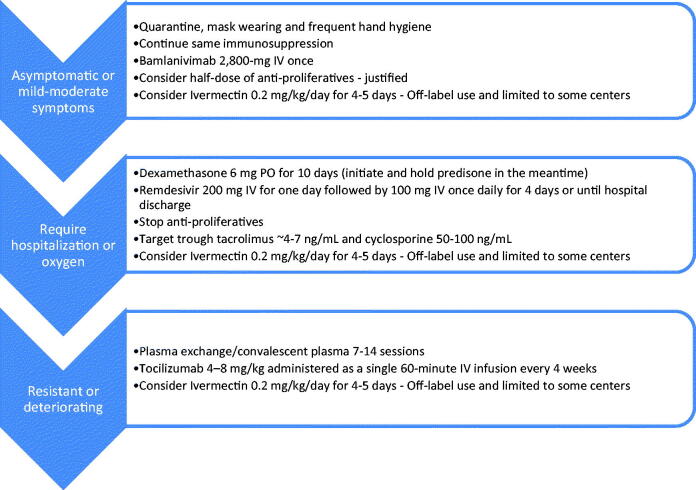
Proposed algorithm for immunosuppressive management in kidney transplant recipients with COVID-19 infection.

The National Health Service (NHS) of England guidelines recommend physicians to exercise caution when starting patients on immunosuppressive medications during the pandemic and to use the lowest possible dose of immunosuppression [46]. The British Society of Rheumatology (BSR) recommends for transplant recipients who are well and on immunosuppression therapy to continue with their medications with appropriate social distancing and infection control measures [66]. Despite theoretical evidence of increased risk of COVID-19 severity and mortality for patients on immunosuppression, to date, various studies have shown immunosuppression per se carry no significant added risk [67,68]. Apparently, major risk factors for severe COVID-19 infection are older age, male gender, obesity and comorbidities of chronic lung disease, diabetes, hypertension or cardiovascular disease. For now, adjusting immunosuppressive medications in transplant candidates is highly dependent on many factors including but not limited to severity of the disease, transplant date and apparent risk of rejection. Current evidence remains scarce and limited to observational and case report studies. A proposed algorithm is illustrated in Figure 1. Most centers adopt reducing or holding antiproliferatives in critically ill patients or in the setting of leukopenia as the first step. Given the possible CNI-anti-COVID effect, many centers keep CNIs at a low dose (target trough levels tacrolimus ∼4–7 ng/mL or cyclosporine A ∼50–100 ng/mL). Based on the RECOVERY trial, dexamethasone is initiated at 6 mg QD PO or IV for 10 days (prednisone may be held in the meantime) [5]. For critically ill patients, the NIH guidelines recommend the addition of remdesivir (to dexamethasone) at a dose of 200 mg IV for one day followed by 100 mg IV once daily for four days or until hospital discharge [46]. Italian and Chinese guidelines have included tocilizumab as a treatment option for selected patients with severe COVID-19 pneumonia [69,70]. Some protocols involve and reserve remdesivir, plasma exchange, convalescent plasma in severe cases [49,55]. Bamlanivimab is FDA authorized and considered for mild-moderate COVID-19 infected cases [51,52]. Ivermectin is used off-label in few centers in the US [48]. Further studies are needed to determine the impact of specific immunosuppressive agents on the course of COVID-19 infection.
